# Conversion of Exogenous Cholesterol into Glycoalkaloids in Potato Shoots, Using Two Methods for Sterol Solubilisation

**DOI:** 10.1371/journal.pone.0082955

**Published:** 2013-12-09

**Authors:** Erik V. Petersson, Nurun Nahar, Paul Dahlin, Anders Broberg, Rikard Tröger, Paresh C. Dutta, Lisbeth Jonsson, Folke Sitbon

**Affiliations:** 1 Department of Plant Biology and Forest Genetics, Uppsala BioCenter, Swedish University of Agricultural Sciences and Linnean Centre for Plant Biology, Uppsala, Sweden; 2 Department of Ecology, Environment and Plant Sciences, Stockholm University, Stockholm, Sweden; 3 Department of Chemistry, Uppsala BioCenter, Swedish University of Agricultural Sciences, Uppsala, Sweden; 4 Chemistry Division 1, Science Department, National Food Agency, Uppsala, Sweden; 5 Department of Food Science, Uppsala BioCenter, Swedish University of Agricultural Sciences, Uppsala, Sweden; Umeå Plant Science Centre, Sweden

## Abstract

Steroidal glycoalkaloids (SGA) are toxic secondary metabolites naturally occurring in the potato, as well as in certain other Solanaceous plant species, such as tomato, eggplant and pepper. To investigate the steroidal origin of SGA biosynthesis, cut potato shoots were fed cholesterol labelled with deuterium (D) in the sterol ring structure (D_5_- or D_6_-labelled), or side chain (D_7_-labelled), and analysed after three or five weeks. The labelled cholesterol and presence of D-labelled SGA were analysed by GC-MS and LC-MS/MS, respectively. When feeding D-labelled cholesterol solubilised in Tween-80, labelled cholesterol in free form became present in both leaves and stems, although the major part was recovered as steryl esters. Minor amounts of D-labelled SGA (α-solanine and α-chaconine) were identified in cholesterol-treated shoots, but not in blank controls, or in shoots fed D_6_-27-hydroxycholesterol. Solubilising the labelled cholesterol in methyl-β-cyclodextrin instead of Tween-80 increased the levels of labelled SGA up to 100-fold, and about 1 mole% of the labelled cholesterol was recovered as labelled SGA in potato leaves. Both side chain and ring structure D labels were retained in SGA, showing that the entire cholesterol molecule is converted to SGA. However, feeding side chain D_7_-labelled cholesterol resulted in D_5_-labelled SGA, indicating that two hydrogen atoms were released during formation of the SGA nitrogen-containing ring system. Feeding with D_7_-sitosterol did not produce any labelled SGA, indicating that cholesterol is a specific SGA precursor. In conclusion, we have demonstrated a superior performance of methyl-β-cyclodextrin for delivery of cholesterol in plant tissue feeding experiments, and given firm evidence for cholesterol as a specific sterol precursor of SGA in potato.

## Introduction

Sterols are major components of cellular membranes in eukaryotic organisms. In animals, cholesterol is the main sterol, whereas plants contain a mixture of sterols with varying length and saturation of the side chain [1]. For a long time, it was believed that plants did not contain cholesterol, but since the finding of cholesterol in potato and *Dioscorea spiculiflora* [2], cholesterol has been found in most plant species, although usually as a minor sterol. However, in some genera, e.g. the Solanaceae and Liliaceae, cholesterol constitutes a significant part of total sterols [3,4]. Besides having a role in membrane structure and function, it has been proposed that plant cholesterol is a precursor of specific steroidal secondary metabolites, such as sapogenins and steroidal glycoalkaloids (SGA) [5]. The steroidal alkaloids are widely spread in Solanum and related genera, including important crop species such as the potato (*Solanum tuberosum*), tomato (*Solanum lycopersicum*) and eggplant (*Solanum melongena*). SGA are not restricted to these families, but are also found in e.g. the Asclepidaceae and Liliaceae. The SGA are toxic [6-9], and food authorities in many countries have established a maximum level of 200 mg total SGA/kg fresh weight (FW) in unpeeled potato tubers aimed for consumption [9,10].

The presence of SGA in potato plants has been known for almost a century [11], but the biosynthetic route has not yet been completely elucidated. According to the suggested biosynthetic pathway, cholesterol is a SGA precursor that via unknown mechanisms is converted to the aglycone solanidine, which is further glycosylated by sequential glycosylation reactions to the two major SGA in commercial potato, α-chaconine and α-solanine [5,7]. The latter steps of this pathway, i.e. the sequential glycosylations starting from solanidine, have been well characterised. Three enzymes and their respective genes have been isolated from potato, and their properties demonstrated *in vitro* and via transgenic plants [12-14]. Also in tomato, the genes responsible for glycosylation of the corresponding aglycone tomatidine have been identified [15,16].

With regard to the early steps of SGA biosynthesis and particularly the role of cholesterol as a precursor, the experimental evidence are however as of yet not fully convincing. Current models are to a large extent based on feeding studies with radioactive precursors in various solanaceous species. In potato, such studies include a report of the incorporation of radioactivity from cholesterol to solanidine [17]. Further, feeding experiments with cholesterol-4-[^14^C] to etiolated potato sprouts indicated that cholesterol was transformed into 26-hydroxycholesterol and cholest-4-en-3-one [18]. Other proposed conversions of cholesterol in Solanaceae have included a hydroxylation at C-26 and the direct replacement of the C-26 hydroxyl group by an amino group [19]. The latter mechanism has been challenged in two recent studies, indicating that the C-26 transamination is occurring via an aldehyde intermediate in tomato [16,20], potato and eggplant [20].

Indirect evidence for cholesterol as the precursor to SGA in potato has come from biochemical analyses of potato tubers, where SGA biosynthesis was initiated after slicing tubers into discs [21]. A regulatory role in SGA biosynthesis was suggested for the sterol methyltransferase enzyme (SMT1), which catalyses the biosynthesis of sterols with an alkylated side-chain (C10-side chain). Inhibition of this enzyme leads to higher amounts of cholesterol (a C8-side chain sterol) [22]. Under conditions where this enzyme was up-regulated and the cholesterol biosynthesis inhibited in the tuber discs, no SGA biosynthesis occurred [23,24]. Further evidence came from transformation studies, where overexpression of the gene for SMT1 in potato resulted in lower amounts of cholesterol and of SGA [25].

**Figure 1:**
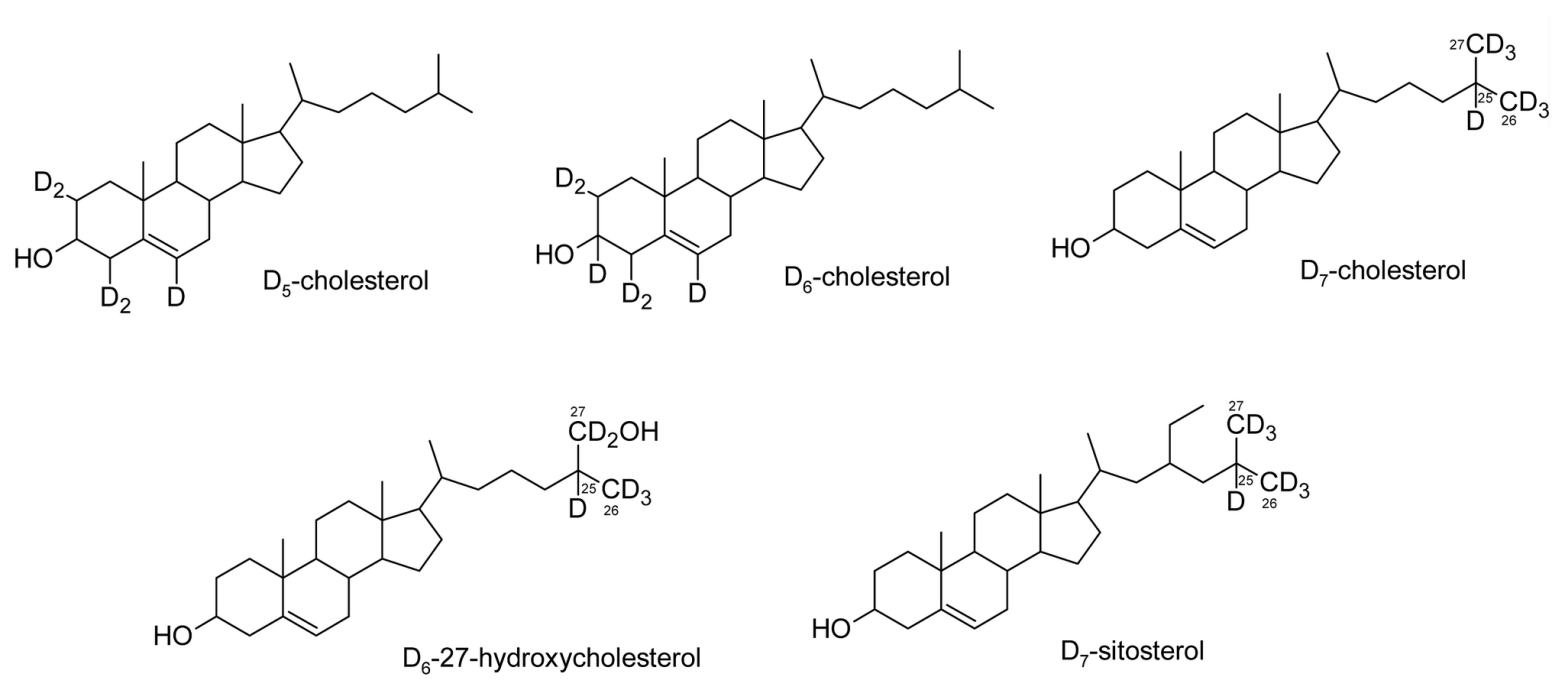
Chemical structures of deuterium (D) -labelled sterols used in the present study. Cut potato shoots were fed D_5_- or D_6_-cholesterol (labelled in the sterol ring structure), or D_7_-cholesterol (labelled in the sterol side chain). Side chain-labelled D_6_-27-hydroxycholesterol and D_7_-sitosterol were used as controls.

The present study aimed at clarifying the role of cholesterol in the biosynthesis of SGA in potato plants. During the publication process, a similar study was published demonstrating side chain-labelled D_6_- and D_7_-cholesterol as SGA precursors in tomato and potato [20]. We here confirm and extend the results concerning the potato SGA metabolism. Using D_5_-, D_6_-, and D_7_-cholesterol, as well as D_6_-27-hydroxycholesterol and D_7_-sitosterol (Figure 1) as substrates, we demonstrate that only cholesterol is incorporated into SGA, and that both the cholesterol ring structure and side chain are retained. Moreover, we show that solubilising deuterated cholesterol in methyl-β-cyclodextrin (MBD) results in a significantly stronger increase in the label incorporation into SGA, compared to the detergent Tween-80. 

## Results and Discussion

### Uptake and distribution of D_5_-labelled cholesterol in potato shoots

**Figure 2:**
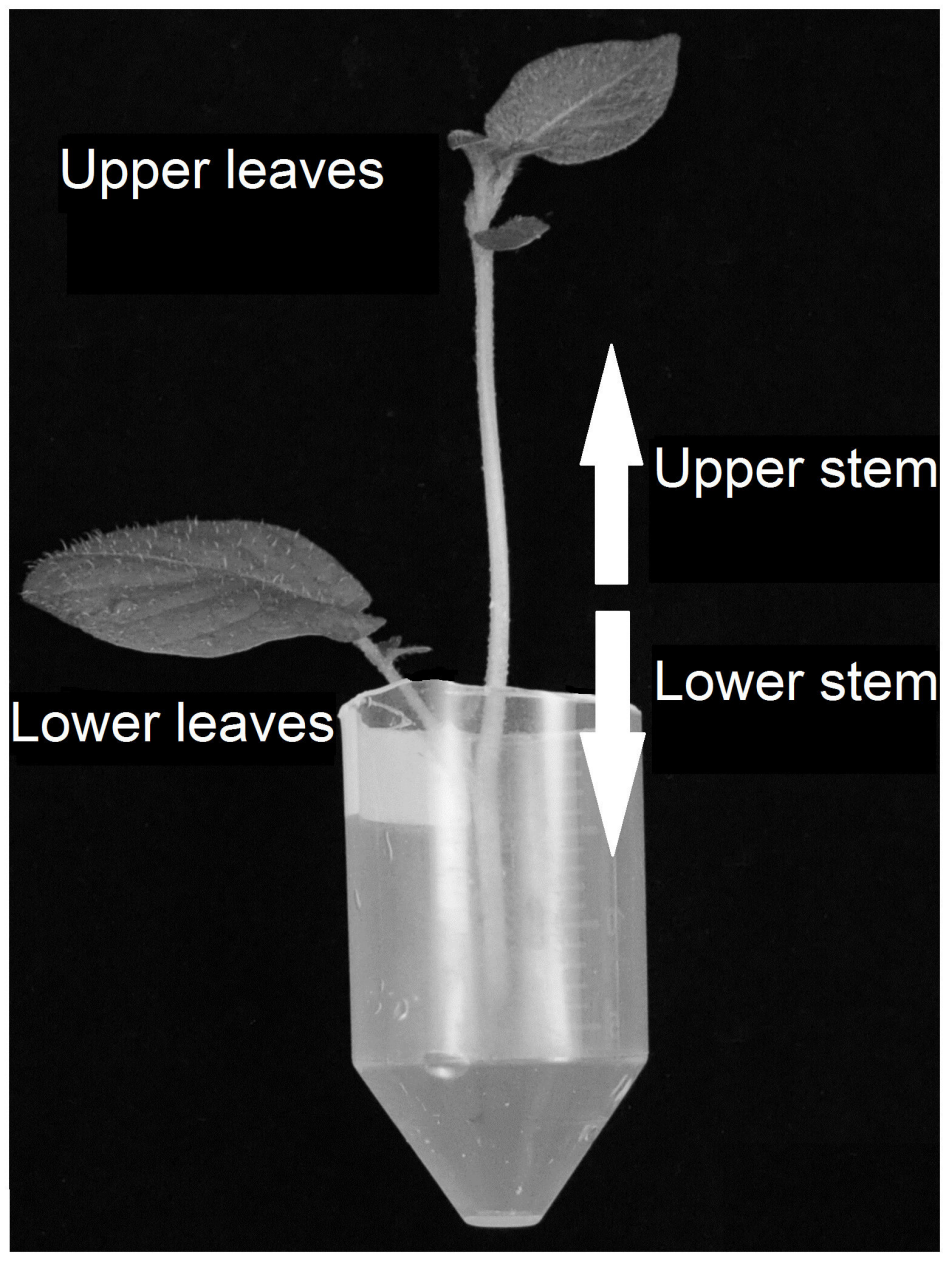
Experimental set-up for sterol feeding studies. Young potato shoots were trimmed to a final length of 10 cm, fed labelled sterols through the cut stem, and incubated for either three or five weeks. After the feeding period, shoots were divided into four parts consisting of upper leaves, lower leaves, upper stem, and lower stem. Materials from up to three separate shoots were pooled when necessary.

Potato shoots (cv. King Edward) were through the cut stem fed 200 µg D_5_-cholesterol in a Tween-80/water suspension (Figure 2). At the end of the incubation period, the general appearance of the shoots was in most cases healthy, although a few shoots showed minor signs of leaf wilting. For mass spectrometry (MS) analysis of metabolites, the shoots were divided into upper and lower leaves and stems (Figure 2). The molecular ions and multiple reaction monitoring (MRM) transitions of both the endogenous and labelled metabolites are described in Table 1. The results showed that all D_5_-cholesterol had been taken up and could be recovered from the shoots, and was distributed in both leaves and the stem (Table 2). A GC-MS chromatogram of labelled and endogenous cholesterol is shown in Figure S1. The main part, ca. 85 % of D_5_-cholesterol, was recovered in the lower stem and in the lower leaves, and ca. 15% was present in the newly developed upper stem and leaves. About 80% of D_5_-cholesterol was recovered after saponification of the sterol fraction, indicating that the major part was stored as steryl esters (Table 2). 

**Table 1:** Molecular ions and fragments monitored for endogenous and deuterium (D) -labelled cholesterol and SGA.

**Compound**	**Molecular ion [M]^*+*^**	**LC-MS/MS: MRM transition to unique fragment ion (*m/z*)**
Cholesterol	386	
D_5_-cholesterol (R)	391	
D_6_-cholesterol (R)	392	
D_7_-cholesterol (SC)	393	
Sitosterol	414	
D_7_-sitosterol (SC)	421	
α-solanine	852	398; 98
α-chaconine	868	398; 98
D_5_-α-chaconine (R)	857	403; 98
D_5_-α-solanine (R)	873	403; 98
D_6_-α-chaconine (R)	858	404; 98
D_6_-α-solanine (R)	874	404; 98
D_5_-α-chaconine (SC)*	857	403; 103
D_5_-α-solanine (SC)*	873	403; 103

Sterols were analysed by GC-MS and SGAs were analysed by LC-MS/MS. Peak identities were verified by comparison to authentic standards. The D labels were either in the ring structure (R), or in the side chain (SC), of the sterols and SGA. Presence (or absence) of side chain label was confirmed in SGA by Multiple Reaction Monitoring (MRM) of mass transitions from the molecular ion to a SC-specific part of the aglycone (e.g. *m/z* 103 or 98 for D_5_-label or no label, respectively). The full aglycone fragment was monitored at *m/z* 398, 403 and 404 (endogenous and labelled).

^*^ formed from D_7_-cholesterol (SC)

**Table 2:** Recovery of D_5_-cholesterol fed to potato shoots.

	**D_5_-cholesterol**		**D_5_-cholesterol released from steryl esters**		**D_5_-SGA**	
	**µg**	**% of total **	**µg**	**% of total**	**µg**	**% of total**
Upper stem	5,1	12	17,5	11	0,05	11
Lower stem	31,2	71	94,9	59	0,02	4
Upper leaves	0,7	2	9,3	6	0,03	7
Lower leaves	6,6	15	39,3	24	0,35	78
Total	43,6	100	161	100	0,45	100

Cut potato shoots (cv. King Edward) were fed 200 µg D_5_-cholesterol solubilised in Tween-80, and sterol and SGA metabolites were analysed after three weeks by GC-MS and LC-MS/MS, respectively. Cholesterol and SGA: n=2 samples per data point (biological duplicate), steryl esters: n=1 sample per data point.

### Metabolism of D_5_-labelled cholesterol in potato shoots

Potato shoots (cv. King Edward), fed as above, or with an equal amount of D_6_-27-hydroxycholesterol (as a control), were analysed for SGA using LC-MS/MS. The *m*/*z* +5 MRM transitions for α-chaconine and α-solanine, showed in the D_5_-cholesterol-treated materials that D_5_-SGA was formed at low levels, but significantly higher than the ^13^C-derived background (Table 3). Similar results were obtained also in cv. Bintje (Table 3), thus confirming the conversion of cholesterol to SGA in another potato cultivar. Since the endogenous SGA was several orders of magnitude higher than the D_5_-SGA, the D_5_-SGA contents were corrected for the natural ^13^C-contribution to the +5 isotopic peaks. Based on the natural abundance of ^13^C, the natural content of the SGA *m*/*z* +5 MRM-transition from the molecular ion to the aglycone was estimated to be 0.0023 % in the blank samples. The evaluation of four control shoots in duplicate showed results quite near this value (α-chaconine 0.0035 % ± 0.0016 %; α-solanine 0.0037 % ± 0.0019 %). After ^13^C-correction, D_5_-SGA levels ranged between 0.01-0.23 mg/kg FW, with the highest levels in lower leaves (Table 3). About 0.1 mole% of the added 200 µg D_5_-cholesterol was recovered in D_5_-SGA when summarized in leaves and stem. Approximately 85% of this D_5_-SGA was located in the leaves (Table 2). By contrast, no increases of labelled SGA could be detected in shoots fed D_6_-27-hydroxycholesterol despite monitoring several possible MRM transitions (not shown). In summary, the results demonstrate that the ring structure of D_5_-cholesterol is incorporated into SGA, and indicate that 27-hydroxycholesterol is not used as precursor for SGA. 

**Table 3:** Endogenous and D_5_-SGA levels in potato shoots fed D_5_-cholesterol.

	**Endogenous SGA (mg/kg FW)**			**D_5_-SGA (mg/kg FW)**		
	**Total SGA**	**α-solanine**	**α-chaconine**	**Total SGA**	**α-solanine**	**α -chaconine**
**cv. 'King Edward' Controls (Blanks)**						
Upper stem	180 (±81)	37 (±18)	143 (±63)	ND	ND	ND
Lower stem	101(±66)	26 (±16)	75 (±50)	ND	ND	ND
Upper leaves	1447(±534)	367 (±152)	1080 (±382)	ND	ND	ND
Lower leaves	546 (±318)	163 (±106)	383 (±212)	ND	ND	ND
**cv. 'King Edward' fed D_5_-cholesterol**						
Upper stem	76 (±3)	15 (±1 )	61 (±2)	0.02 (±0.00)	0.00	0.02 (±0.00)
Lower stem	72 (±3)	22 (±1 )	50 (±2)	0.02 (±0.00)	0.00	0.02 (±0.00)
Upper leaves	1539 (±311)	378 (±73)	1161 (±238)	0.05 (±0.01)	0.01 (±0.00)	0.04 (±0.01)
Lower leaves	308 (±100)	77 (±25)	231 (±75)	0.23 (±0.02)	0.03 (±0.01)	0.20 (±0.02)
**cv. 'Bintje' Controls (Blanks)**						
Upper stem	106	39	67	ND	ND	ND
Lower stem	63	22	41	ND	ND	ND
Upper leaves	467	139	328	ND	ND	ND
Lower leaves	67	17	50	ND	ND	ND
**cv. 'Bintje' fed D_5_-cholesterol**						
Upper stem	194	71	124	0.01	0.00	0.01
Lower stem	256	106	150	0.02	0.00	0.02
Upper leaves	377	98	279	0.13	0.01	0.12
Lower leaves	188	39	149	0.15	0.02	0.13

Cut potato shoots (cvs. King Edward and Bintje) were fed 200 µg D_5_-cholesterol solubilised in Tween-80, and SGAs were analysed after three weeks by LC-MS/MS. Mean value ± range of measurements in biological duplicates (n=2 samples) for cv. King Edward, and a single analysis for cv. Bintje (n=1 sample). ND = not detected.

### Levels of endogenous SGA and sterols in shoots fed D_5_-labelled cholesterol

Addition of significant amounts of cholesterol might be anticipated to perturb the normal metabolism of sterols and SGA. Hence, as a control, the effects of added D_5_-cholesterol on SGA and sterol levels were studied in the different tissues.

Endogenous SGA levels in both cv. King Edward and cv. Bintje were generally highest in the upper, young leaves followed by the lower, older leaves (Table 3). For all the tissues investigated, the endogenous level of SGA, and the ratio between α-chaconine and α-solanine, was similar in control shoots and shoots fed D_5_-cholesterol. 

To study the effects of D_5_-cholesterol feeding on sterol levels, both cholesterol and the other three major desmethylsterols in potato plants: sitosterol, campesterol and stigmasterol, were analysed in cv. King Edward (Table 4). Compared to blank controls, the shoots that had been fed D_5_-cholesterol had similar levels of free cholesterol, and a similar ratio between endogenous cholesterol and sitosterol, stigmasterol, or campesterol. For obvious reasons, the shoots that had been fed D_5_-cholesterol contained much higher cholesterol levels as compared to the control shoots. Earlier studies on mutants overproducing sterols have shown that plant cells are able to accommodate high amounts of sterols as steryl esters in lipid particles [26,27]. The main part of the added D_5_-cholesterol was indeed recovered in esterified form, distributed similarly to its free form, i.e. the highest levels were found in the older parts of the plants, the lower stem and the lower leaves (Table 4). The ratios between esterified and free cholesterol in the different tissues were similar for the D-labelled and the endogenous species. Still, when considering only the free cholesterol, the shoots fed D_5_-cholesterol had in all tissues higher amounts of total cholesterol (endogenous and D_5_-cholesterol) than the control plants. This raises the question of how the shoots managed to maintain membrane function. Since the general appearance of the treated shoots was not different from controls, we suggest that the sterol composition in the membranes was kept stable and that the added D_5_-cholesterol became localised in a different subcellular pool compared to the endogenous sterols. It has recently been shown in tobacco and Chinese cabbage, that cholesterol is the major sterol in the phloem exudate [28]. It is also of interest that one of the enzymes in sterol biosynthesis, Δ7-sterol-C_5_-desaturase, was found localised to lipid droplets which were clearly seen in vascular tissue [29]. It is tempting to speculate that the additional amounts of cholesterol in the D_5_-cholesterol-fed shoots occur in a transport form, possibly in the phloem sap. Nevertheless, the results demonstrate that the sterol feeding process is not associated with an alteration of the endogenous metabolism of sterols or SGA. 

**Table 4:** Sterol levels in potato shoots fed D_5_-cholesterol.

	**Sitosterol (mg/kg FW)**		**Campesterol (mg/kg FW)**		**Stigmasterol (mg/kg FW)**		**Cholesterol (mg/kg FW)**		**D_5_-cholesterol (mg/kg FW)**	
	**FS**	**SE**	**FS**	**SE**	**FS**	**SE**	**FS**	**SE**	**FS**	**SE**
**Controls (Blanks**)										
Upper stem	8.8 (±5.2)	69	2.6 (±1.5)	20	0.6 (±0.3)	3.8	1.2 (±0.2)	6.7	ND	ND
Lower stem	6.0 (±1.9)	18	1.3 (±0.6)	3.3	0.4 (±0.0)	1.1	0.5 (±0.1)	1.2	ND	ND
Upper leaves	12.0 (±6.9)	70	5.6 (±3.5)	31	0.7 (±0.3)	4.6	3.0 (±1.9)	14	ND	ND
Lower leaves	14.0 (±3.1)	76	9.0 (±1.1)	30	1.0 (±0.3)	3.4	2.8 (±0.2)	10	ND	ND
**Fed D_5_-cholesterol**										
Upper stem	6.5 (±3.1)	38	2.7 (±1.8)	14	0.4 (±0.1)	1.3	1.3 (±0.3)	4.3	4.5 (±4.1)	17
Lower stem	9.3 (±0,6)	36	2.7 (±0.9)	9.8	0.6 (±0.0)	4.6	1.0 (±0.1)	4.9	42 (±31)	126
Upper leaves	12.0 (±0.5)	123	7.3 (±0.3)	37	0.7 (±0.2)	9.1	3.7 (±0.0)	35	2.1 (±0.7)	29
Lower leaves	11.0 (±4.1)	88	7.5 (±2.3)	25	0.5 (±0.1)	4.6	3.6 (±0.5)	20	9.3 (±0.7)	51

Cut potato shoots (cv. King Edward) were fed 200 µg D_5_-cholesterol solubilised in Tween-80, and desmethylsterol levels were analysed after three weeks using GC-MS. Mean value ± range of measurements in biological duplicates (n=2 samples), or single analysis for conjugated sterols (n=1 sample). FS, free sterol; SE, steryl esters; ND, not detected.

### Increased conversion of cholesterol to SGA by solubilisation in methyl-β-cyclodextrin

The results from feeding D_5_-cholesterol solubilised in Tween-80 suggested that the D_5_-cholesterol was not available for conversion into SGA to the same extent as endogenous cholesterol. In order to investigate this aspect, we carried out a feeding experiment in cv. King Edward comparing cholesterol solubilised in Tween-80 or in methyl-β-cyclodextrin (MBD), another substance commonly used for sterol solubilisation. In this experiment, both D_6_(ring)- and D_7_(side chain)-labelled cholesterol were used for two main reasons. The first reason is that the natural ^13^C-contribution to the formed D-SGA is lower for heavier substrates, such as D_6_-cholesterol, and the second reason is that the side chain-labelled cholesterol gives additional information about the fate of the side chain (Figure 1). Water blanks were used as controls. As an additional control, feeding was also carried out with D_7_-labelled sitosterol, to investigate whether conversion to SGA is specific for cholesterol. All samples were monitored for endogenous as well as *m*/*z* +5, +6, and +7 MRM-transitions (Table 1).

**Figure 3:**
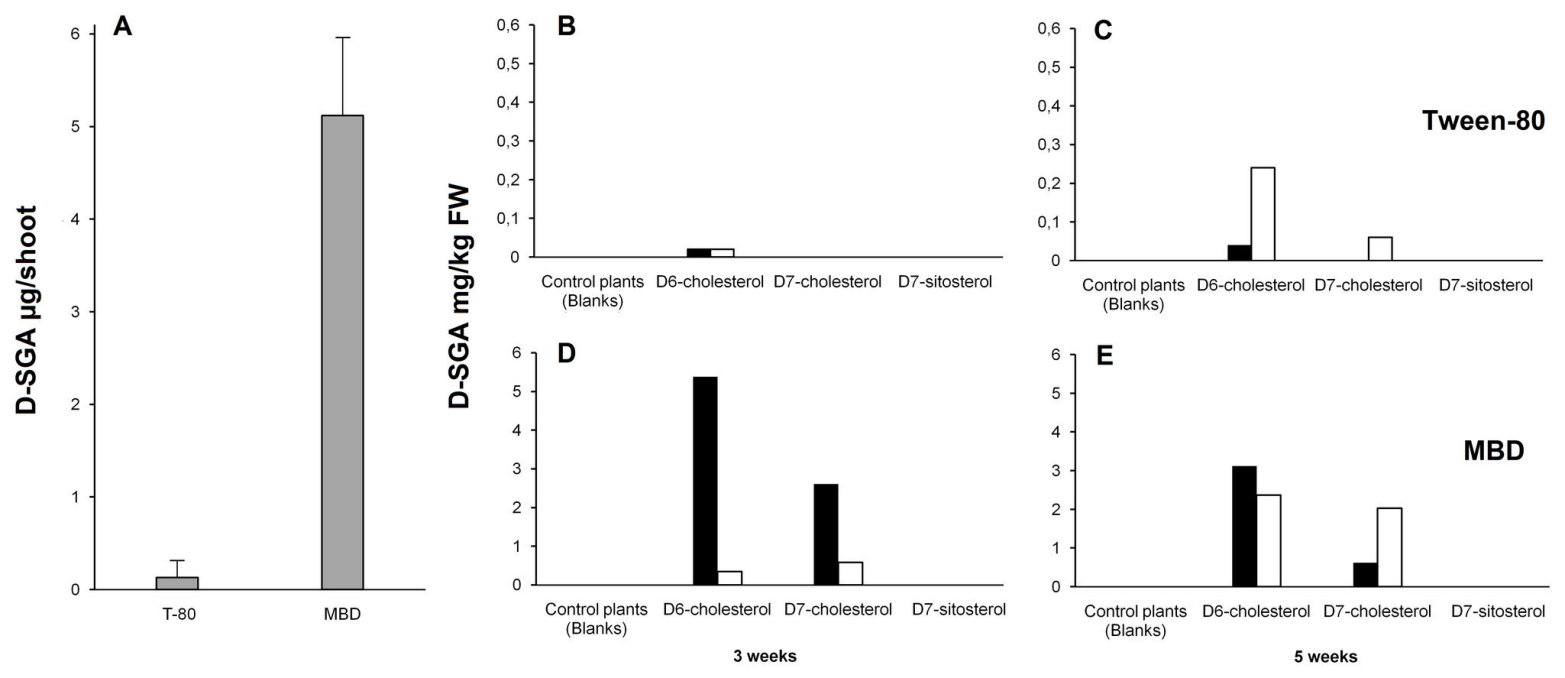
Formation of deuterium (D) -labelled SGA in potato shoots after feeding with D-labelled sterols solubilised in Tween-80 or methyl-β-cyclodextrin (MBD). Cut potato shoots (cv. King Edward) were fed 200 µg D_6_- cholesterol, D_7_-cholesterol, or D_7_-sitosterol solubilised in Tween-80 or MBD, and incubated during either three or five weeks. After the incubation period, the levels of D-SGA (α-solanine and α-chaconine) were analysed in upper leaves (black bars) and lower leaves (white bars) using LC-MS/MS. (A) Graphical illustration of the difference between Tween-80 (T-80) and MBD feedings. Mean value ± SD for the amount of D-SGA formed in leaves up to 5 weeks. A difference between the treatments (n=4 full plants each) was significant at p<0.001 (paired Student's *t*-test). (B) to (E) individual analyses, each representing a single analysis of materials from 2-3 pooled plants: (B) Tween-80, 3 weeks; (C) Tween-80, 5 weeks; (D) MBD, 3 weeks; (E) MBD, 5 weeks. Note the different scales for Tween-80 and MBD. No D-SGA was formed in parallel analyses of blank controls, or shoots fed D_7_-sitosterol.

Endogenous and D-labelled SGA were analysed in the upper and lower leaves after 3 and 5 weeks (Table S1). A representative LC-MS/MS chromatogram is shown in Figure S2. When comparing all samples, significantly more D-SGA was formed with MBD (p< 0.001; two-tailed, paired Student's *t*-test) as shown in Figure 3A. MBD samples had an average amount of D-SGA formed of 5.12 µg/shoot; about 40 times higher than the average in Tween-80 samples (0.13 µg/shoot). For individual samples, the levels of labelled SGA in shoots fed D_6_-cholesterol or D_7_-cholesterol increased up to 100-fold when cholesterol was administered in MBD, as compared to Tween-80 (Figure 3B-E). 

For Tween-80 samples, the shoots fed D-labelled cholesterol during 5 weeks had slightly higher levels D-SGA than the shoots fed during 3 weeks. This difference was not observed in MBD samples. For all shoots, the levels of D-labelled SGA tended to decrease with time in the upper leaves and increase in the lower leaves. Slightly higher amounts of D-labelled SGA were formed from D_6_-cholesterol compared to D_7_-cholesterol (Table 5). When D-labelled cholesterol was administered in MBD, the absolute amounts of labelled SGA equalled or were higher than those of labelled free cholesterol (Table 5). In the leaves of plants fed D_6_-cholesterol or D_7_-cholesterol, ca. 1-3 mole% of the added 200 µg D-labelled cholesterol was recovered in free cholesterol, and typically 1 mole% in SGA.

**Table 5:** Deuterated (D) SGA and sterols in potato shoots fed D-labelled cholesterol or sitosterol solubilised in methyl-β-cyclodextrin (MBD).

	**Endogenous sterol**		**Deuterium-labelled sterol**		**Deuterium-labelled SGA**	
**Sterol fed**	**3 weeks µg/shoot**	**5 weeks µg/shoot**	**3 weeks µg/shoot**	**5 weeks µg/shoot**	**3 weeks µg/shoot**	**5 weeks µg/shoot**
D_6_-cholesterol	8.7	8.5	3.4	3.0	5.7	6.0
D_7_-cholesterol	7.6	22.4	1.5	6.2	4.5	4.3
D_7_-sitosterol	37.2	22.6	0.8	1.1	ND	ND

Cut potato shoots (cv. King Edward) were fed 200 µg D-labelled cholesterol or sitosterol solubilised in MBD, grown for either three or five weeks, after which sterols and SGA were analysed by GC-MS and LC-MS/MS, respectively. The amount per shoot is calculated from mean values of a single analysis of upper and lower leaves, respectively, that were pooled from 2-3 independent plants. ND, not detected.

In shoots fed D_7_-sitosterol, the recovery of D_7_-sitosterol in the leaf tissue was somewhat lower than the corresponding recovery in shoots fed D_6_-, and D_7_-cholesterol (Table 5), possibly reflecting a lower solubility or transport rate for sitosterol in MBD as compared to cholesterol. No SGA of any D-label was detected in these shoots, indicating that sitosterol is not converted into SGA (Figure 3B-E, Table 5, Table S1). 

**Figure 4:**
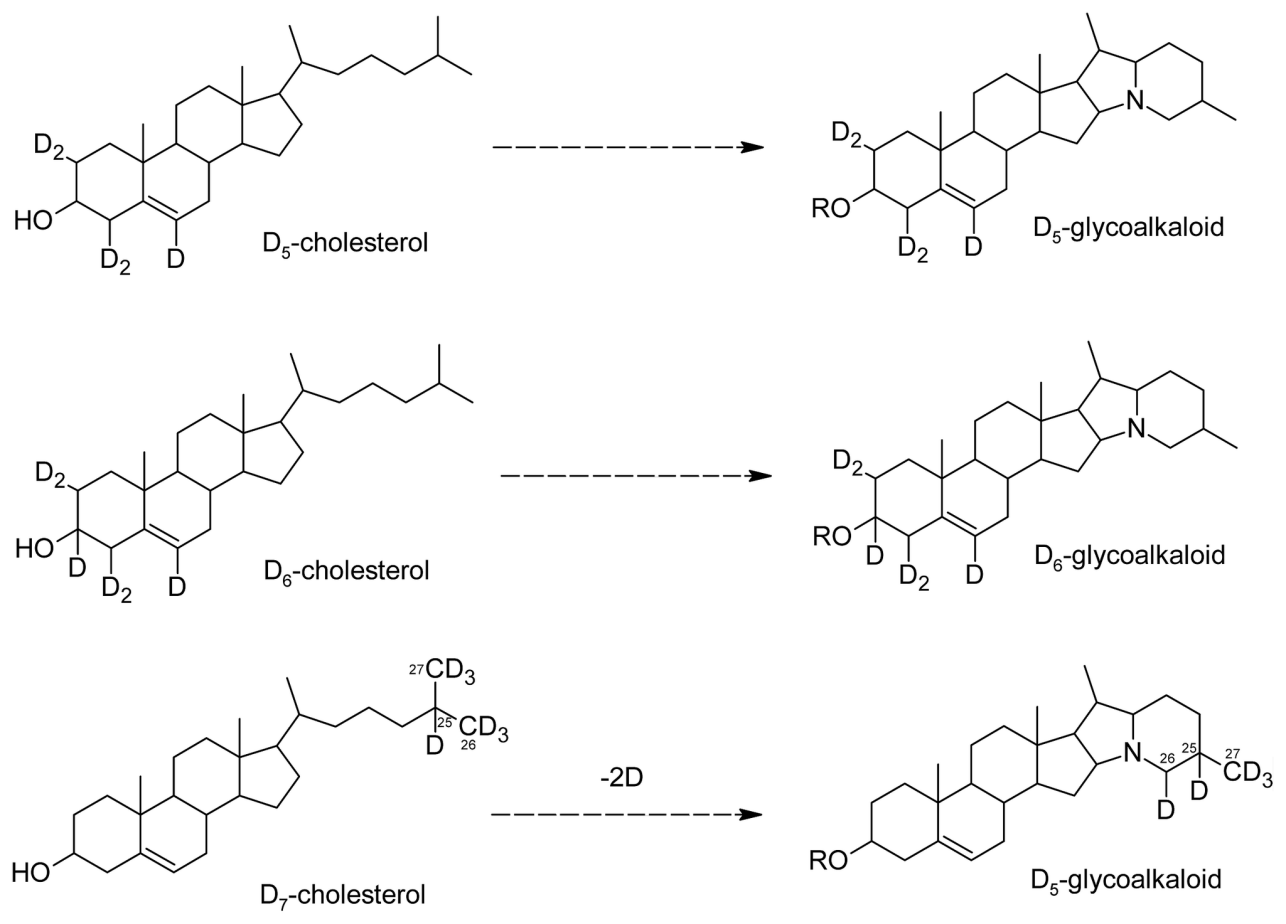
A model for the conversion of different deuterium (D) -labelled cholesterol substrates to D-labelled glycoalkaloids. Cut potato shoots were fed D_5_- or D_6_-cholesterol (labelled in the sterol ring structure), or D_7_-cholesterol (labelled in the sterol side chain). All these labels were converted *in* *vivo* to D-labelled SGA. Labels in the ring structure were intact after conversion to SGA, but two D atoms were lost from the side-chain label, indicative of an aldehyde formation, likely at the C-26 position. R signifies either OH (solanidine), chacotriose (α-chaconine), or solatriose (α-solanine).

As expected, it was found that the ring-labelled D_6_-cholesterol formed D_6_-SGA. However, the side-chain labelled D_7_-cholesterol formed D_5_-SGA (*m*/*z* +5), both regarding α-solanine and α-chaconine, rather than the expected D_7_-SGA (*m*/*z* +7). This means that two hydrogen atoms from the side chain are detached during the closure of the nitrogen-containing ring system, and confirms recent results showing loss of two hydrogen atoms from the C-26 position during ring closure [20]. Moreover, our results show that the hydrogen loss is specific to the cholesterol side chain, and do not occur from the A+B rings. The conversion of different cholesterol substrates to their presumed end products is outlined in Figure 4.

One factor worth considering is that levels of endogenous SGA are several orders of magnitude higher than the formed D_5_- and D_6_-SGA. The natural ^13^C-contribution to the *m*/*z* +6 MRM is only 0.0001%, and thereby negligible. On the other hand, for the formed D_5_-SGA, the ^13^C-contribution to the *m*/*z* +5 MRM is 0.0023% and could make a significant contribution to this peak. However, shoots fed D_7_-cholesterol in MBD formed D_5_-SGA well above what is significantly affected by ^13^C-contribution. This contrasts to some extent to the incorporation of D_5_-cholesterol using Tween-80 (Table 3), where lower levels of D_5_-SGA were formed, and thus required correction. 

### Analysis of hydroxysterol intermediates

One of the suggested intermediates in the biosynthesis of SGA from cholesterol is 26-hydroxycholesterol [18,20]. Compared to what is known about the metabolism of plant sterols, considerably less is known about that of hydroxysterols. However, recent studies have clearly demonstrated hydroxysterols as endogenous sterol metabolites in plants [16,30]. Using GC-MS and authentic hydroxysterol standards, we identified 26-hydroxycholesterol (Figure S3) as well as 22-hydroxycholesterol as endogenous hydroxysterols in leaves of potato, but not in Arabidopsis, a species lacking SGA (Table S2). Thus, formation of 26-hydroxycholesterol may be an initial step in potato SGA biosynthesis. However, an attempt to detect D-labelled 26-hydroxycholesterol was negative, although this may be explained by the low endogenous 26-hydroxycholesterol levels (0.18 mg/kg FW). Based on the proportions between the endogenous intermediate and end product, it seems reasonable to assume that substantial amounts of fed leaf material will be required for analysis of labelled intermediates. More research is needed to optimise hydroxysterol labelling conditions in vivo, and the analytical methods, to be able to investigate whether the isotopic enrichment in the pool of specific hydroxysterols is consistent with a precursor role in SGA biosynthesis.

Given the structure required to close the N-containing ring system in solanidine (fused cyclic amine structure) compared to that in tomatidine (oxo-aza spiro structure), we speculate that 22-hydroxycholesterol is an endogenous hydroxysterol in potato, but not an important intermediate in the biosynthesis of potato SGA. The suggested 22,26-dihydroxycholesterol intermediate found in tomato [16] could not be investigated due to lack of adequate standards. We presume that also this compound is more relevant in tomatine biosynthesis. Based on these considerations, a model for biosynthesis of potato SGA from cholesterol is shown in Figure S4.

### Choice of solubilisation method

Collectively, our results demonstrate a specific conversion of D-labelled cholesterol into D-labelled SGA, with retention of both the cholesterol side chain and ring structure labels. Moreover, solubilising cholesterol in MBD instead of Tween-80, renders the sterol more available for conversion into SGA.Tween-80 is a nonionic surfactant (i.e. without ionic interaction) and emulsifier, consisting of a hydrophilic and a lipophilic group. As a surface-active agent, it has the potential to form a micellar structure at or above a concentration threshold known as the critical micelle concentration in solution. In micelles, the hydrophobic parts of a surfactant molecule associate to form a core region, whereas the hydrophilic parts position between the core and the external aqueous medium. In this way, the hydrophobic core gets stabilised by the hydrophilic shell [31]. Cyclodextrins, on the other hand, are cyclic oligosaccharides composed of a hydrophilic outer surface and a lipophilic central cavity that have the capacity to bind lipophilic compounds in their hydrophobic part [32]. The water-soluble MBD can form soluble inclusion complexes with cholesterol, thereby enhancing its solubility in aqueous solution. Our results show that forming an inclusion complex of cholesterol with MBD results in a much higher conversion rate of D-labelled cholesterol to SGA. This may be due to a more efficient uptake and transport in combination with a more suitable availability for enzymes in the following steps in the conversion to SGA. 

## Conclusions

An initial challenge was to solubilise a lipophilic compound, cholesterol, in a solution that would facilitate uptake and metabolism in plant tissues. We demonstrate here that the choice of solubilisation method has a profound impact on the conversion of cholesterol into SGA in cut potato shoots. When forming an inclusion complex of cholesterol with MBD, cholesterol is up to 100-fold more efficiently converted to SGA, than when forming micelles with Tween-80. In the leaves of plants fed D_6_-cholesterol or D_7_-cholesterol solubilised with MBD, ca. 1-3 mole% of the added D-labelled cholesterol (200 µg) was recovered in free cholesterol, and typically 1 mole% in SGA. Feeding side chain D_7_-labelled cholesterol resulted in D_5_-labelled SGA, indicating that two hydrogen atoms are released from the side chain during closure of the nitrogen-containing ring system. Feeding with D_7_-sitosterol or D_6_-27-hydroxycholesterol did not produce detectable amounts of labelled SGA. In conclusion, we have demonstrated a superior performance of MBD for the delivery of cholesterol in plant tissue feeding experiments, and given firm evidence for cholesterol as a specific precursor of SGA in potato. Our results confirm and extend previous studies that cholesterol constitutes an SGA precursor in potato plants [5,20], and can now be used as a base for further metabolic studies of SGA as well as other sterol-derived secondary metabolites in potato and other plant species.

## Materials and Methods

### Plant materials, and feeding conditions

Potato tubers of cvs. ‘King Edward’ and 'Bintje' were stored in the dark for two weeks at 20 °C to induce sprouting. The tubers were grown on soil in a growth chamber kept at 22 °C, and received white fluorescent light at a fluence rate of 110 µmoles m^-2^ s^-1^ during a 16 h photoperiod. Shoots 15-20 cm long were harvested and trimmed to 10 cm by cutting off the base stem. The shoots were then weighed and placed into plastic tubes with tap water for three days to recover from any wounding stress (Figure 2). Solutions with D_5_-cholesterol (A, B-ring labelled; 2,2,4,4,6-D_5_; Medical Isotopes Inc, Pelham, NH, USA) and D_6_-27-hydroxycholesterol (side-chain labelled; 25,26,26,26,27,27-D_6_, Avanti Polar Lipids Inc., Alabaster, AL, USA) were prepared by dissolving the sterols in 99.5% ethanol containing 5 mg Tween-80 (Riedel-de Haën, Hannover, Germany) and drying under nitrogen gas. The solids were then re-dissolved in 100 µl water containing 2% ethanol, and diluted to 1 ml with water. Tubes with 200 μg solubilised sterol was used for the labelling experiment, while the tubes for the control plants (blanks) were filled with a commercial fertiliser solution (Blomstra, Cederoth AB, Upplands Väsby, Sweden). All plants were grown in a growth chamber under the same growth condition as mentioned above. When the sterol solution (or fertiliser solution) after 3 to 5 days had been taken up, the tubes were refilled with additional fertiliser solution until three weeks in total had passed. 

To form complexes with methyl-β-cyclodextrin (MBD), labelled sterols were dissolved following the protocol of Klein et al. [33], slightly modified by adding 400 µl isopropanol instead of chloroform. Exactly 200 μg of dissolved sterols, corresponding to 74 μl of the labelled sterol-MBD mixture, was diluted with autoclaved water to a final volume of 1 ml, which was fed to the cut shoots. D_6_-cholesterol (A,B-ring labelled; 2,2,3,4,4,6-D_6_; Cambridge Isotopes, Andover, MA, USA), D_7_-cholesterol (sidechain-labelled; 25,26,26,26,27,27,27-D_7_; Medical Isotopes Inc, Pelham, NH, USA), or D_7_-sitosterol (side chain-labelled; 25,26,26,26,27,27,27-D_7_; Medical Isotopes Inc, Pelham, NH, USA) were added to shoots prepared as above. Blank control plants were fed only tap water. In parallel, the same amount of D_6_-cholesterol, D_7_-cholesterol and D_7_-sitosterol was dissolved in Tween-80 as described earlier and fed to separate shoots. After uptake of the sterol solution, tap water was added during seven days to prevent any interaction between nutrients and the sterol-MBD complex. The following weeks, fertiliser solution suspended in tap water (1:1000) was added to the shoots. Blank controls were treated the same way. 

At the end of the feeding experiments (i.e. after 3 or 5 weeks), shoots were quickly rinsed with tap water, and divided into four parts consisting of upper and lower leaves; as well as upper and lower stems (Figure 2), and frozen in liquid nitrogen. When necessary, pools of up to three separate shoots were made to obtain sufficient materials for analysis.

### Sterol extraction and subsequent analysis with GC-MS

Free 4-desmethylsterols were analysed in leaf and stem samples of ‘King Edward‘ shoots. In brief, samples were homogenised in a mortar using liquid nitrogen, transferred to a glass tube, followed by addition of 43 µg desmosterol (Sigma-Aldrich, Schnelldorf, Germany) as internal standard (IS). Total lipids were extracted with 5 ml chloroform:methanol (2:1, v/v) for 1 h at 70 °C. When sterols released from conjugated forms were measured, an additional saponification step was applied [25]. The sterol-containing chloroform phase was removed, dried under nitrogen gas, and reconstituted in 3 ml hexane. The extracts were purified with a solid phase extraction using ISOLUTE® Si 500 mg/6 ml cartridges (Biotage, Uppsala, Sweden) and the following procedure: activation with 3 ml hexane, loading the sample (in 3 ml hexane), washing with 9 ml hexane, eluting with 3 ml hexane:ethyl acetate (50:50 v/v). The eluate was dried under nitrogen gas and reconstituted in 1 ml hexane. The 4-desmethylsterols were analysed by GC-MS and quantified relative to the IS. Standards used for sterol identification were: endogenous cholesterol (Sigma-Aldrich, Schnelldorf, Germany); 2,2,4,4,6-D_5_-cholesterol, 25,26,26,26,27,27,27-D_7_-cholesterol, 25,26,26,26,27,27,27-D_7_-sitosterol (all from Medical Isotopes Inc, Pelham, NH, USA); 2,2,3,4,4,6-D_6_-cholesterol (Cambridge Isotopes, Andover, MA, USA); as well as the other major 4-desmethylsterols. 

Sterols were characterised and identified by GC-MS. Free sterol analysis was performed on a GC8000 Top Series gas chromatograph (CE Instruments, Milan; Italy) with a DB35-MS column (15 m x 0.20 mm, 0.33 µm film thickness) from J&W Scientific (J&W Scientific, Folsom, CA, USA) coupled to a Voyager mass spectrometer (Finnigan, Manchester, UK). Injector temperature was 260 °C, and oven conditions were 60 °C for 1 min and raised to 270 °C at rate of 30 °C/min and maintained for 2 min and then raised to 290 °C at a rate of 1 °C/min. The temperatures of the transfer line to the MS and the detector were set to 290 °C. Helium was used as carrier gas at a constant pressure of 80 kPa. The samples were injected (1 µl) in split mode (ca. 50:1). Full scan mass spectra (*m/z* 50-450) were recorded at 70 eV. Both endogenous and labelled sterols were quantified by their total ion count (TIC) against the IS.

Hydroxycholesterols were extracted and purified using 19-hydroxycholesterol as an internal standard according to Beste et al. [30], with some modifications. The starting size of the sample was 20 grams of leaves, both upper and lower, in order to get enough material for quantification. The sample was extracted and concentrated throughout the procedure to a final volume of 400 µl TMSi derivative dissolved in hexane, which was analysed using GC-MS on a HP5890 GC (Hewlett-Packard, Palo Alto, CA, USA) equipped with an HP-5MS column (30 m x 0.25 mm, 0.25 µm film thickness) connected to a HP5970 MSD. Injector temperature was 260 °C, and oven conditions were 60 °C for 1 min and raised to 290 °C at rate of 50 °C/min and maintained for 10 min and then raised to 300 °C at rate of 1 °C/min and maintained for 15 min. The temperatures of the transfer line to the MS and the detector were set to 300 °C. Helium was used as carrier gas at a constant flow of 1 ml/min. The samples were injected (1 µl) in splitless mode. Hydroxysterols were analysed (at 70 eV) in selected ion monitoring (SIM) mode for increased sensitivity, and quantified against the internal standard using the major ion fragments for the hydroxycholesterol standards (*m/z* 546; 456; 417; 366; 353; 173; 145; 131; 129; 83; 73). Corresponding ion fragments for labelled counterparts were also monitored in samples fed labelled cholesterol. The standards used for comparisons were: 19-hydroxycholesterol, 20α-hydroxycholesterol, 22(R)-hydroxycholesterol (Research Plus Inc., Barnegat, NJ, USA), and 26-hydroxycholesterol (Avanti Polar Lipids Inc., Alabaster, AL, USA).

### Glycoalkaloid extraction and analysis with LC-MS/MS and LC-UV

SGA were extracted from leaves and stems and purified according to Petersson et al. [34]. Solamargine was during the initial extraction step added as an internal standard, corresponding to 100 µg/g (FW) potato tissue. A mass ratio of 1 part sample and 4 parts of extraction liquid was used. The LC-MS/MS equipment used was an Agilent Technologies 6410 Triple Quadrupole with an Agilent 1200 Series HPLC system (Agilent Technologies, Waldbronn, Germany) equipped with a Synergi Fusion-RP column (4 µm, 2.1 mm x 75 mm) from Phenomenex (Torrance, CA, USA). The mobile phase was 22 % acetonitrile acidified with 0.1 % formic acid, and the flow rate was 0.5 ml/min. The mass spectrometer was used with a source voltage of 4 kV. MRM transitions monitored are shown in Table 1, and the collision energies used to fragment the molecular ions of α-solanine and α-chaconine (both endogenous and labelled) were 85 eV (to fragments *m/z* 398; 403; 404) and 95 eV (to fragments *m/z* 98; 103). 

Endogenous SGA levels were quantified using LC-UV rather than LC-MS/MS, since the UV response was linear over a greater range of concentrations, and endogenous SGA levels were high enough for this less sensitive technique. After quantification with LC-UV, the levels of the labelled SGA were calculated by their relative LC-MS/MS response within the same sample, which proved to be constant and unaffected by ion suppression. The LC-UV equipment was an HP 1100 series (Hewlett Packard, Waldbronn, Germany) with a G1312A binary pump and a G1314A UV-detector set to 202 nm. Separation was achieved with a Hypersil Gold C18 column (5 µm, 2.1 mm x 150 mm) from Thermo Scientific (Waltham, MA, USA) using a mobile phase consisting of 10 mM phosphate buffer pH 7.6 with 36 % acetonitrile, at a flow rate of 0.5 ml/min. The standards used for the analyses were α-chaconine 95% (Sigma-Aldrich, Schnelldorf, Germany), α-solanine 95% (MP Biomedicals, Solon, OH, USA) and solamargine >98 % (Glycomix, Reading, UK).

## Supporting Information

Figure S1 **GC-MS chromatogram from an analysis of endogenous- and deuterium (D) -labelled cholesterol in potato leaves.** Cut potato shoots (cv. King Edward) were fed 200 µg D_5_-cholesterol solubilised in Tween-80, after which leaves were analysed for endogenous and D_5_-cholesterol by GC-MS. The chromatogram shows the molecular ions (M^+^) of D_5_-cholesterol and endogenous cholesterol extracted from the total ion current during the same run.
(PDF)

Figure S2 **LC-MS/MS chromatogram from an analysis of potato leaves containing deuterium (D) -labelled SGA.** Cut potato shoots (cv. King Edward) were fed 200 µg D_6_-cholesterol solubilised in methyl-β-cyclodextrin after, which leaves were analysed for endogenous and D-SGA by LC-MS/MS. (A) D_6_-α-solanine (left peak) and D_6_-α-chaconine (right peak); and (B) endogenous α-solanine (left peak) and endogenous α-chaconine (right peak).
(PDF)

Figure S3 **GC-MS chromatogram from an analysis of hydroxysterols in potato leaves.** Endogenous 26-hydroxycholesterol (retention time 43 min) in a potato shoot (cv. King Edward) analysed with GC-MS in single ion monitoring (SIM) mode. The peak matched an authentic 26-hydroxycholesterol standard both regarding retention time and characteristic ion fragments.
(PDF)

Figure S4 **Working model for the SGA biosynthesis from cholesterol.** The reaction scheme is freely adapted from [16,20]. Occurrence of 26-hydroxycholesterol as an endogenous hydroxysterol in potato plants was demonstrated in the present study.
(PDF)

Table S1 **Levels of endogenous and deuterium (**D**) -labelled SGA.** The SGA metabolites indicated in the table were analysed in upper and lower leaves of potato shoots (cv. King Edward) that had been fed 200 µg D-labelled sterols solubilised in Tween-80 or in methyl-β-cyclodextrin (MBD) for three or five weeks, and compared to water control samples (blanks). Each data point is based on one analysis of an extract made from two or three pooled shoots from independent plants. Levels of D-labelled SGA are corrected for the natural abundance of ^13^C where needed. ND, not detected.
(PDF)

Table S2 **Hydroxysterol levels in leaves of potato and Arabidopsis.** Endogenous hydroxysterols were at two separate occasions measured in leaves of potato (cv. King Edward) and Arabidopsis (cv. Columbia), using 19-hydroxycholesterol as internal standard and quantification by GC-MS. Mean value ± range (n=2 separate analyses). ND, not detected.
(PDF)
