# Supplementary material for: Conversion of Exogenous Cholesterol into Glycoalkaloids in Potato Shoots, Using Two Methods for Sterol Solubilisation
Source: PLoS One. 2013 Dec 9;8(12):e82955. doi: 10.1371/journal.pone.0082955 (PMC3857313; doi:10.1371/journal.pone.0082955)

Figure S1. GC-MS chromatogram from an analysis of endogenous- and deuterium (D) -labelled cholesterol in potato leaves.
Cut potato shoots (cv. King Edward) were fed 200 µg D_5-cholesterol solubilised in Tween-80, after which leaves were analysed for endogenous and D_5-cholesterol by GC-MS. The chromatogram shows the molecular ions (M^+) of D_5-cholesterol and endogenous cholesterol extracted from the total ion current during the same run.

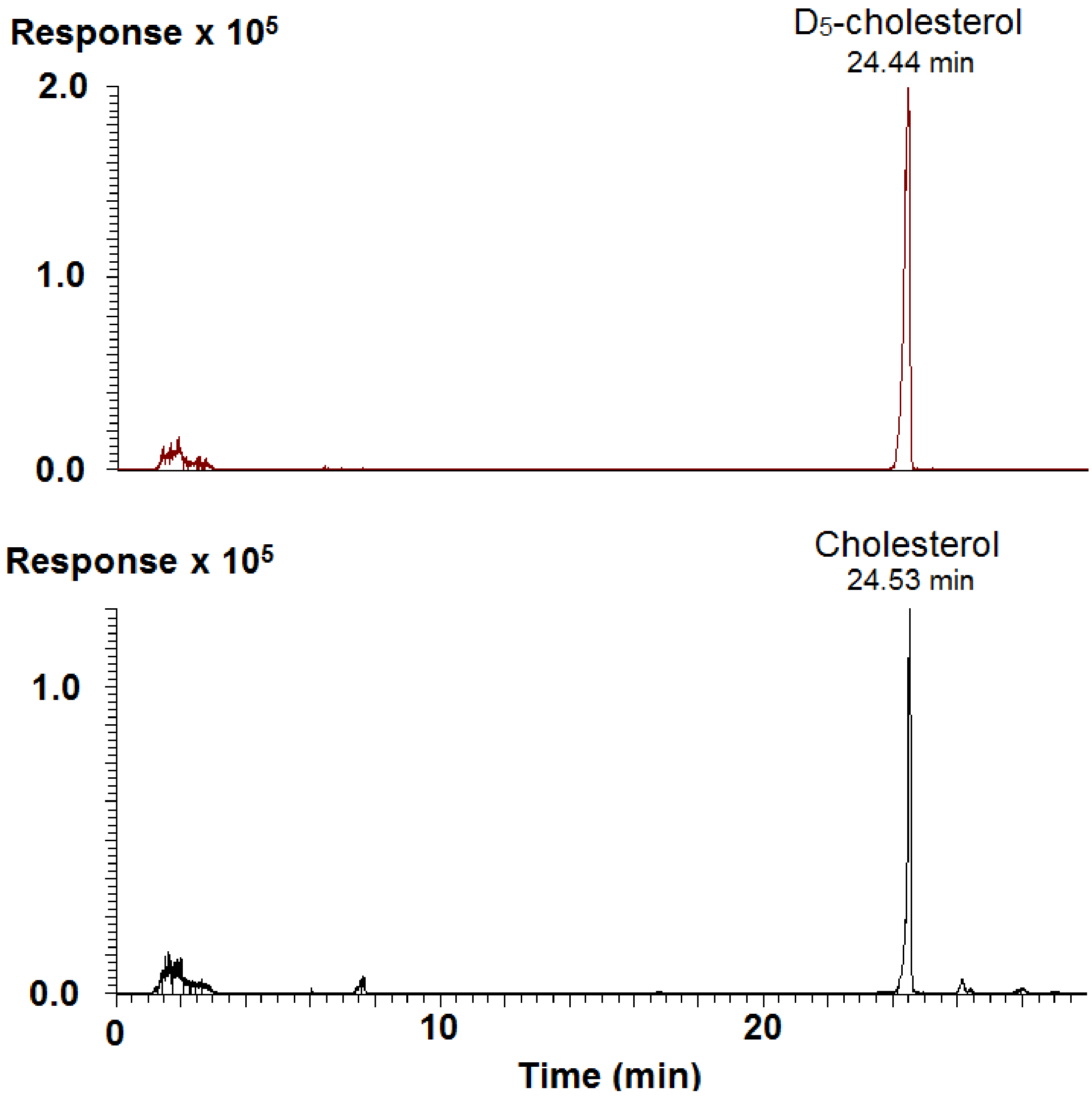

Supplement: Figure S1 — GC-MS chromatogram from an analysis of endogenous- and deuterium (D) -labelled cholesterol in potato leaves. Cut potato shoots (cv. King Edward) were fed 200 µg D5-cholesterol solubilised in Tween-80, after which leaves were analysed for endogenous and D5-cholesterol by GC-MS. The chromatogram shows the molecular ions (M+) of D5-cholesterol and endogenous cholesterol extracted from the total ion current during the same run. (PDF) [file pone.0082955.s001.pdf]
