# Supplementary material for: Conversion of Exogenous Cholesterol into Glycoalkaloids in Potato Shoots, Using Two Methods for Sterol Solubilisation
Source: PLoS One. 2013 Dec 9;8(12):e82955. doi: 10.1371/journal.pone.0082955 (PMC3857313; doi:10.1371/journal.pone.0082955)

Figure S2. LC-MS/MS chromatogram from an analysis of potato leaves containing deuterium (D) -labelled SGA.
Cut potato shoots (cv. King Edward) were fed 200 µg D_6-cholesterol solubilised in methyl-β-cyclodextrin after, which leaves were analysed for endogenous and D-SGA by LC-MS/MS. (A) D_6-α-solanine (left peak) and D_6-α-chaconine (right peak); and (B) endogenous α-solanine (left peak) and endogenous α-chaconine (right peak).

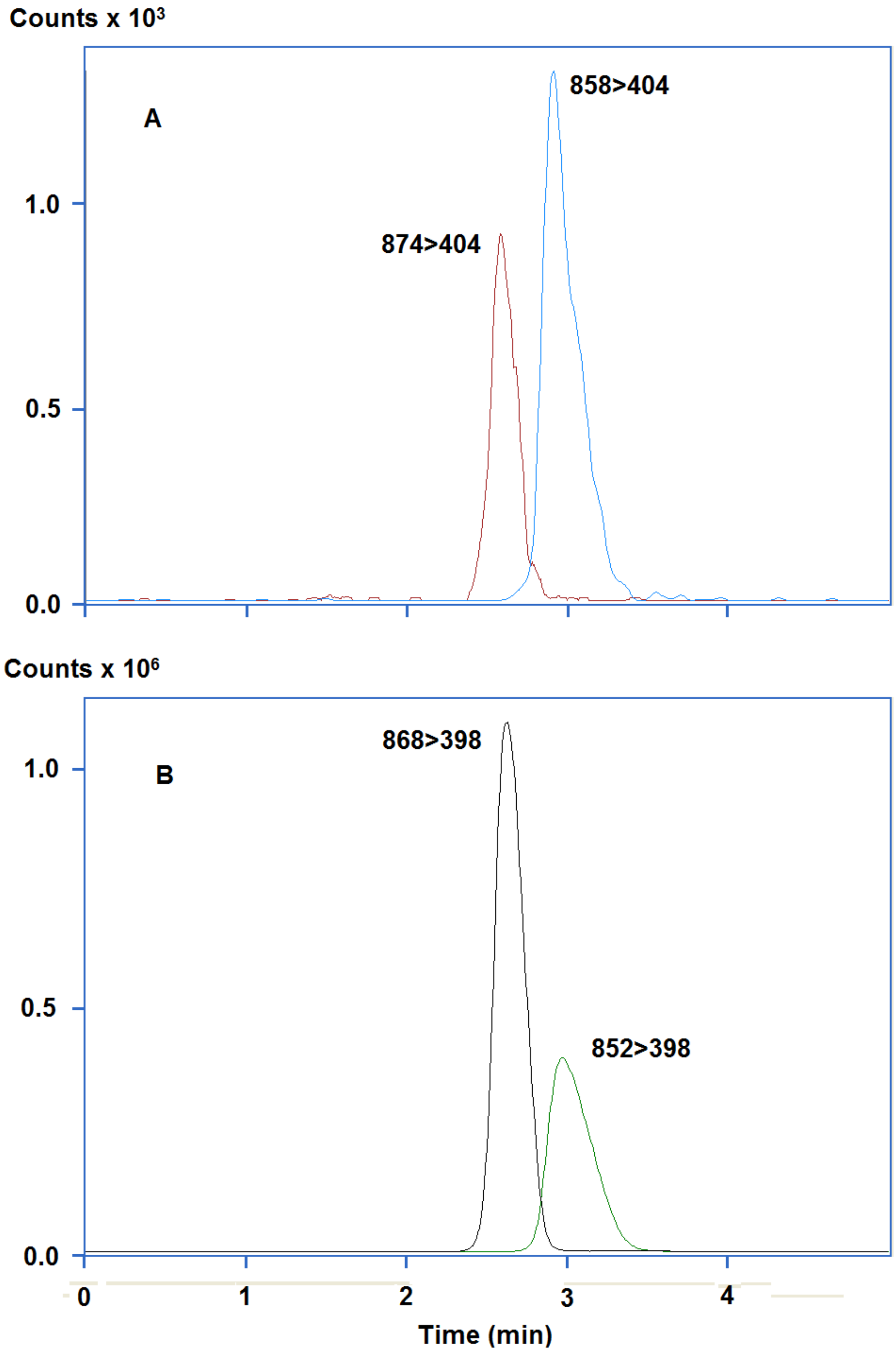

Supplement: Figure S2 — LC-MS/MS chromatogram from an analysis of potato leaves containing deuterium (D) -labelled SGA. Cut potato shoots (cv. King Edward) were fed 200 µg D6-cholesterol solubilised in methyl-β-cyclodextrin after, which leaves were analysed for endogenous and D-SGA by LC-MS/MS. (A) D6-α-solanine (left peak) and D6-α-chaconine (right peak); and (B) endogenous α-solanine (left peak) and endogenous α-chaconine (right peak). (PDF) [file pone.0082955.s002.pdf]
