# Supplementary material for: Conversion of Exogenous Cholesterol into Glycoalkaloids in Potato Shoots, Using Two Methods for Sterol Solubilisation
Source: PLoS One. 2013 Dec 9;8(12):e82955. doi: 10.1371/journal.pone.0082955 (PMC3857313; doi:10.1371/journal.pone.0082955)

Figure S3. GC-MS chromatogram from an analysis of hydroxysterols in potato leaves.
Endogenous 26-hydroxycholesterol (retention time 43 min) in a potato shoot (cv. King Edward) analysed with GC-MS in single ion monitoring (SIM) mode. The peak matched an authentic 26-hydroxycholesterol standard both regarding retention time and characteristic ion fragments.

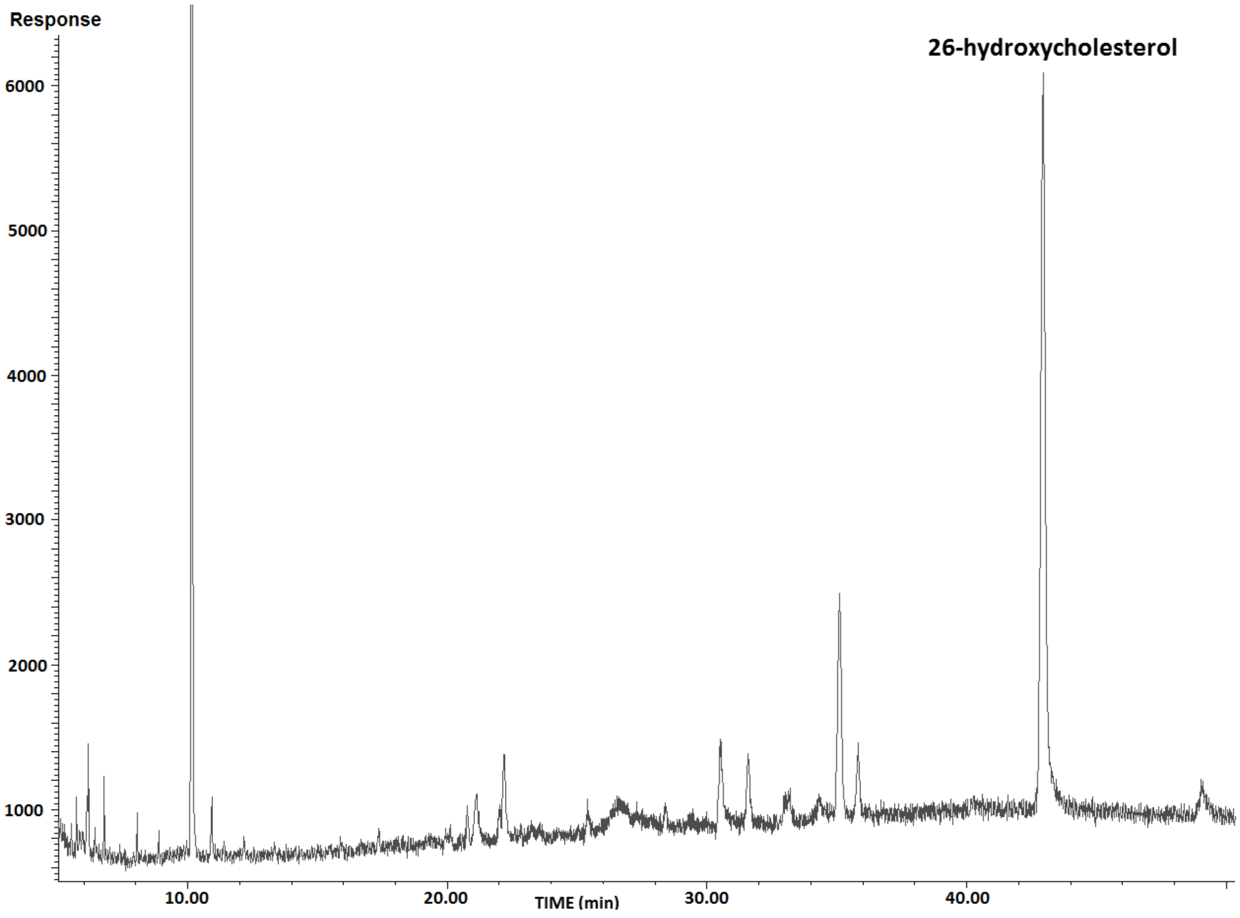

Supplement: Figure S3 — GC-MS chromatogram from an analysis of hydroxysterols in potato leaves. Endogenous 26-hydroxycholesterol (retention time 43 min) in a potato shoot (cv. King Edward) analysed with GC-MS in single ion monitoring (SIM) mode. The peak matched an authentic 26-hydroxycholesterol standard both regarding retention time and characteristic ion fragments. (PDF) [file pone.0082955.s003.pdf]
