# Supplementary material for: Conversion of Exogenous Cholesterol into Glycoalkaloids in Potato Shoots, Using Two Methods for Sterol Solubilisation
Source: PLoS One. 2013 Dec 9;8(12):e82955. doi: 10.1371/journal.pone.0082955 (PMC3857313; doi:10.1371/journal.pone.0082955)

Figure S4. Working model for the SGA biosynthesis from cholesterol.
The reaction scheme is freely adapted from [16,20]. Occurrence of 26-hydroxycholesterol as an endogenous hydroxysterol in potato plants was demonstrated in the present study.

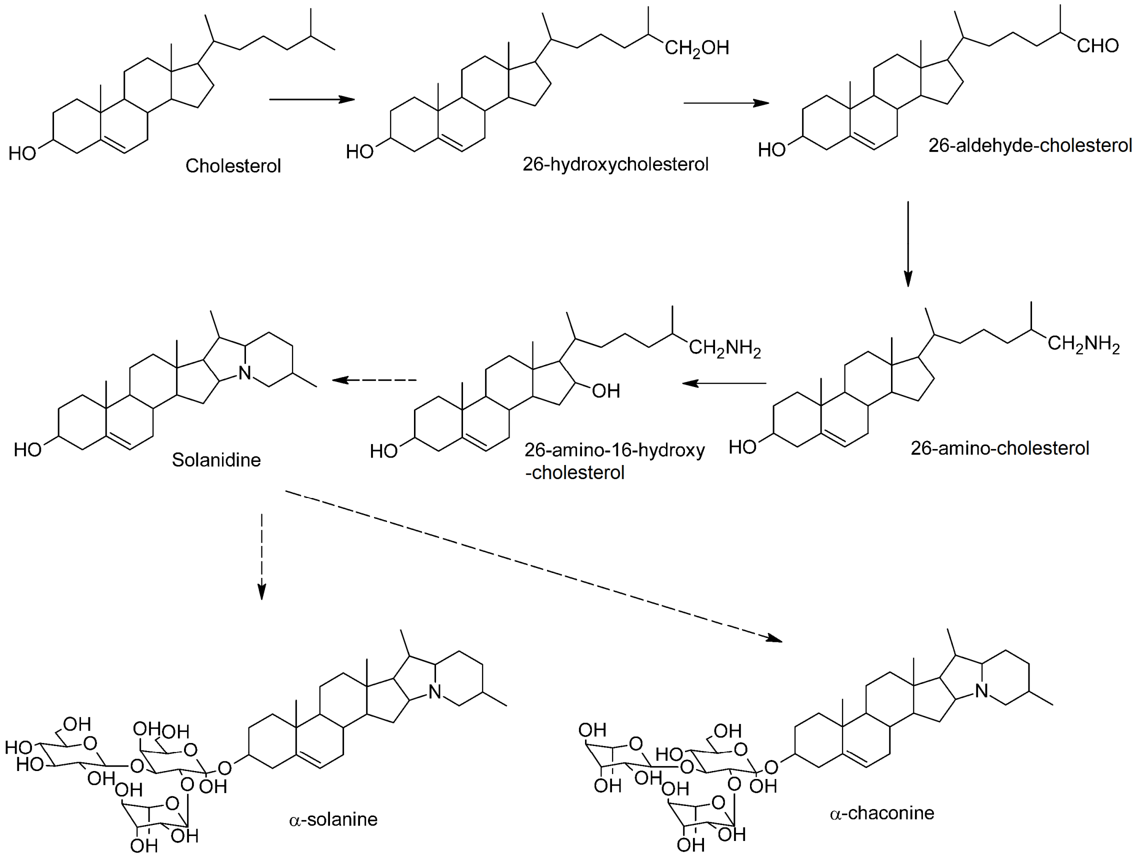

Supplement: Figure S4 — Working model for the SGA biosynthesis from cholesterol. The reaction scheme is freely adapted from [16,20]. Occurrence of 26-hydroxycholesterol as an endogenous hydroxysterol in potato plants was demonstrated in the present study. (PDF) [file pone.0082955.s004.pdf]
