# Supplementary material for: Conversion of Exogenous Cholesterol into Glycoalkaloids in Potato Shoots, Using Two Methods for Sterol Solubilisation
Source: PLoS One. 2013 Dec 9;8(12):e82955. doi: 10.1371/journal.pone.0082955 (PMC3857313; doi:10.1371/journal.pone.0082955)
Supplement: Table S1 — Levels of endogenous and deuterium (D) -labelled SGA. The SGA metabolites indicated in the table were analysed in upper and lower leaves of potato shoots (cv. King Edward) that had been fed 200 µg D-labelled sterols solubilised in Tween-80 or in methyl-β-cyclodextrin (MBD) for three or five weeks, and compared to water control samples (blanks). Each data point is based on one analysis of an extract made from two or three pooled shoots from independent plants. Levels of D-labelled SGA are corrected for the natural abundance of 13C where needed. ND, not detected. (PDF) [file pone.0082955.s005.pdf]

**Table S1. Levels of endogenous and deuterium (D) -labelled SGA.**
The SGA metabolites indicated in the table were analysed in upper and lower leaves of potato shoots (cv. King Edward) that had been fed 200 µg D-labelled sterols solubilised in Tween-80 or in methyl-β-cyclodextrin (MBD) for three or five weeks, and compared to water control samples (blanks). Each data point is based on one analysis of an extract made from two or three pooled shoots from independent plants. Levels of D-labelled SGA are corrected for the natural abundance of $^{13}C$ where needed. ND, not detected.

| | | | **3 weeks (mg/kg FW)** | | | | **5 weeks (mg/kg FW)** | | | |
|---|---|---|---|---|---|---|---|---|---|---|
| **Solubiliser** | **Sterol fed** | **Shoot part** | α-solanine endogenous | α-chaconine endogenous | α-solanine $D_5$-$D_7$ | α-chaconine $D_5$-$D_7$ | α-solanine endogenous | α-chaconine endogenous | α-solanine $D_5$-$D_7$ | α-chaconine $D_5$-$D_7$ |
| **Tween-80** | **Control (Blank)** | upper leaves | 1088 | 2024 | ND | ND | 901 | 2343 | ND | ND |
| | | lower leaves | 425 | 636 | ND | ND | 477 | 779 | ND | ND |
| | **$D_6$-cholesterol** | upper leaves | 1109 | 2372 | ND | 0,02 | 1227 | 2952 | 0,01 | 0,03 |
| | | lower leaves | 495 | 842 | ND | 0,02 | 701 | 1277 | 0,02 | 0,22 |
| | **$D_7$-cholesterol** | upper leaves | 1250 | 2618 | ND | ND | 1023 | 2638 | ND | ND |
| | | lower leaves | 388 | 656 | ND | ND | 1143 | 1873 | ND | 0,06 |
| | **$D_7$-sitosterol** | upper leaves | 838 | 1898 | ND | ND | 1440 | 3290 | ND | ND |
| | | lower leaves | 489 | 863 | ND | ND | 780 | 1382 | ND | ND |
| **MBD** | **Control (Blank)** | upper leaves | 1088 | 2024 | ND | ND | 901 | 2343 | ND | ND |
| | | lower leaves | 425 | 636 | ND | ND | 477 | 779 | ND | ND |
| | **$D_6$-cholesterol** | upper leaves | 952 | 1848 | 0,69 | 4,68 | 1018 | 2385 | 0,28 | 2,85 |
| | | lower leaves | 518 | 825 | 0,04 | 0,32 | 832 | 1407 | 0,31 | 2,06 |
| | **$D_7$-cholesterol** | upper leaves | 903 | 1959 | 0,34 | 2,26 | 946 | 2437 | 0,11 | 0,51 |
| | | lower leaves | 379 | 729 | 0,08 | 0,51 | 1193 | 1948 | 0,32 | 1,71 |
| | **$D_7$-sitosterol** | upper leaves | 1122 | 1743 | ND | ND | 1373 | 3153 | ND | ND |
| | | lower leaves | 448 | 733 | ND | ND | 1279 | 2345 | ND | ND |
