# Supplementary material for: Conversion of Exogenous Cholesterol into Glycoalkaloids in Potato Shoots, Using Two Methods for Sterol Solubilisation
Source: PLoS One. 2013 Dec 9;8(12):e82955. doi: 10.1371/journal.pone.0082955 (PMC3857313; doi:10.1371/journal.pone.0082955)
Supplement: Table S2 — Hydroxysterol levels in leaves of potato and Arabidopsis. Endogenous hydroxysterols were at two separate occasions measured in leaves of potato (cv. King Edward) and Arabidopsis (cv. Columbia), using 19-hydroxycholesterol as internal standard and quantification by GC-MS. Mean value ± range (n=2 separate analyses). ND, not detected. (PDF) [file pone.0082955.s006.pdf]

Table S2. Hydroxysterol levels in leaves of potato and Arabidopsis. Endogenous hydroxysterols were at two separate occasions measured in leaves of potato (cv. King Edward) and Arabidopsis (cv. Columbia), using 19-hydroxycholesterol as internal standard and quantification by GC-MS. Mean value ± range (n=2 separate analyses). ND, not detected.

Compound	Potato mg/kg FW	Arabidopsis mg/kg FW
20-hydroxycholesterol	ND	ND
22-hydroxycholesterol	0.20 (±0.08)	ND
26-hydroxycholesterol	0.18 (±0.14)	ND
